# Role of hydrogen sulfide in bacterial antibiotic tolerance: from mechanisms to therapeutic targeting

**DOI:** 10.3389/fcimb.2026.1875957

**Published:** 2026-07-30

**Authors:** Fengshan Li, Jiawen Shen, Huiru Cao, Xiaoqing Wang, Yulu Du, Yalong Liu, Tianyu Dong, Hua Tian, Salwa E. Gomaa, Xianghui Li, Guanbin Qi, Chaonan Ma

**Affiliations:** 1 Department of Pulmonary and Critical Care Medicine, Huaihe Hospital of Henan University, Henan University, Kaifeng, China; 2 Henan International Joint Laboratory for Nuclear Protein Regulation, School of Basic Medical Sciences, Henan University, Kaifeng, Henan, China; 3 Department of Pulmonary and Critical Care Medicine, the First Affiliated Hospital of Henan University, Kaifeng, China; 4 Department of Microbiology and Immunology, Faculty of Pharmacy, Zagazig University, Zagazig, Egypt; 5 Oncology Department, Kaifeng Central Hospital, Kaifeng, China

**Keywords:** antibiotic resistance, bacterial persisters, H_2_S biosynthesis inhibitors, hydrogen sulfide (H_2_S), multidrug tolerance mechanisms

## Abstract

Hydrogen sulfide (H_2_S) is fundamentally a gaseous signaling molecule in bacteria. Recent studies have reported that H_2_S is a key mediator of bacterial antibiotic tolerance, making it a promising strategic target for overcoming tolerance, eliminating persisters, and restoring antibiotic efficacy. We systematically review recent advances in H_2_S-mediated multidrug tolerance mechanisms, highlighting three core pathways: iron chelation for antioxidant defense, reactive sulfur species-mediated antibiotic inactivation and efflux pump activation, and metabolic dormancy induced by respiratory inhibition, and note that recent advances in H_2_S fluorescent probes have provided important tools for monitoring intracellular H_2_S changes in bacteria under antibiotic stress. Furthermore, this review systematically summarizes recent advances in the development of novel inhibitors targeting intracellular H_2_S biosynthesis in bacteria.

## 1 Introduction

Antimicrobial resistance is a major global health threat, causing millions of deaths each year and forcing clinicians to rely on last-resort antibiotics like carbapenems and polymyxins for severe infections such as sepsis, pneumonia, and postoperative cases (Reza et al., 2025). Among bacterial resistance mechanisms, the toxin-antitoxin (TA) system and SOS response mediate dormancy and DNA repair, but targeting them as downstream nodes is limited by redundant pathway compensation and strong selective pressure (Rafiq et al., 2025; Ravikumar and Kurthkoti, 2026). Studies have shown, endogenous H_2_S weakens antibiotic killing through multiple parallel actions—antioxidation, iron homeostasis and respiratory interference (Selvakumar et al., 2024). Its biosynthetic enzymes are highly conserved in pathogens but differ in hosts, allowing specific small-molecule inhibitors to break resistance, restore bactericidal activity with low resistance risk, and enhance immune clearance. Therefore, as an upstream regulatory hub, H_2_S offers greater clinical translational potential than the TA system and SOS response.

**Figure 1:**
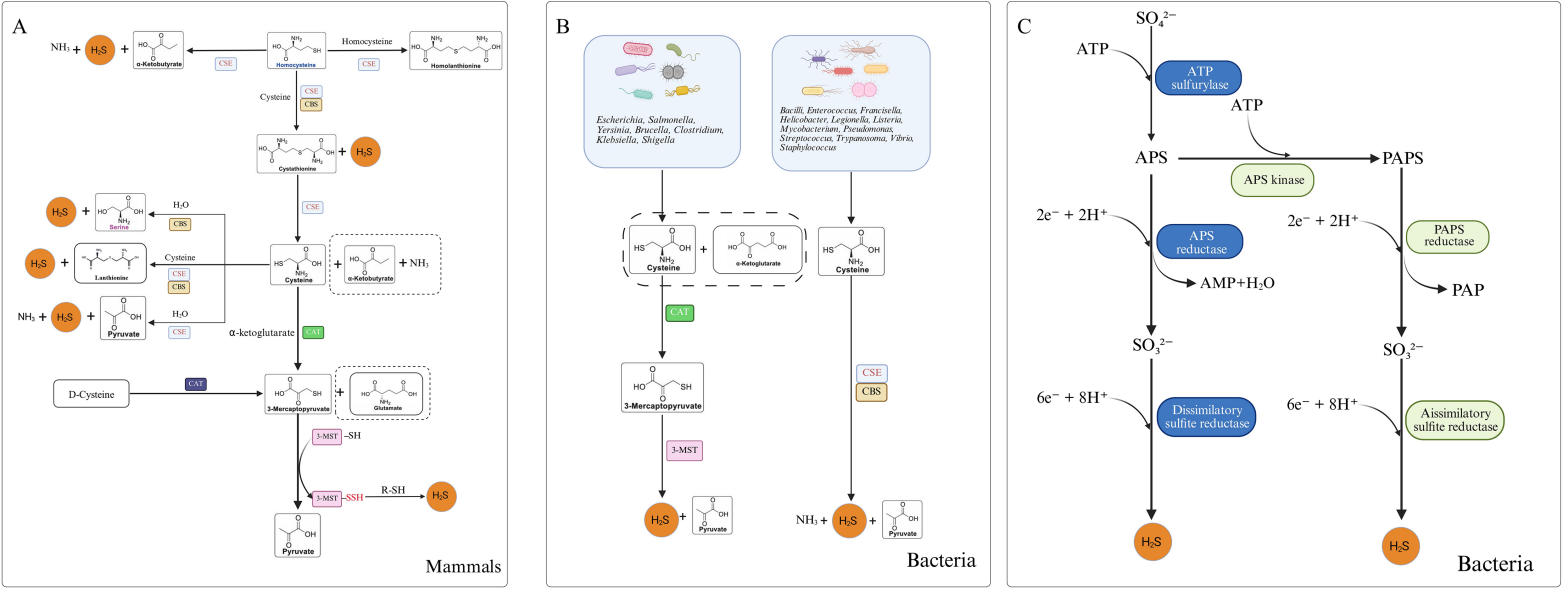
Endogenous H_2_S biosynthesis pathways in mammals and bacteria. **(A)** Mammals synthesize H_2_S primarily via three enzymes: CBS, CSE, and 3-MST. CBS and CSE produce H_2_S through the metabolism of various sulfur-containing amino acids, whereas 3-MST generates H_2_S via a CAT-dependent cascade reaction using D-cysteine as a substrate. **(B)** In many bacteria, such as Gram-positive Bacillus species and certain Gram-negative strains, H_2_S is directly produced from cysteine by enzymes homologous to CBS and CSE. In contrast, within Enterobacteriaceae (e.g., *E. coli*), 3-MST homologs catalyze H_2_S generation through the 3-mercaptopyruvate pathway. **(C)** Sulfate-reducing bacteria utilize a distinct, multi-step pathway for H_2_S production. This process involves ATP-dependent sulfate activation to form APS or PAPS, followed by sulfite generation catalyzed by APS reductase or PAPS reductase, and ultimately the conversion of sulfite to H_2_S via sulfite reductase.

H_2_S is recognized as one of the endogenous gasotransmitters alongside oxide (NO) and carbon monoxide (CO), plays a pivotal role in physiological processes such as the maintenance of cardiovascular homeostasis, regulation of neural function, and modulation of immune responses (Ding et al., 2022; Cirino et al., 2023). In mammals, H_2_S is primarily generated through the catalysis of sulfur-containing amino acids by three key enzymes: cystathionine-β-synthase (CBS), cystathionine-γ-lyase (CSE), and 3-mercaptopyruvate sulfurtransferase (3-MST) (Zhang et al., 2021; Rao et al., 2023; Corvino and Caliendo, 2024) (**Figure 1A**). In contrast, H_2_S has traditionally been viewed as a byproduct of sulfur metabolism, and its physiological functions, particularly in non-sulfur bacteria, remained elusive until recently (Seregina et al., 2022). Recent studies reveal that bacteria share conserved enzymatic machinery (CBS, CSE, and 3-MST homologs) with mammals for the biosynthesis of H_2_S (**Figures 1B, C**). Despite structural similarities with mammalian enzymes, the bacterial homologs demonstrate distinct specificity in their physiological functions, ecological significance, and biosynthetic regulatory mechanisms (Luhachack and Nudler, 2014; Kimura, 2015; Szabo, 2018).

In clinically important pathogens such as *S. aureus*, *P. aeruginosa*, and *B. anthracis*, the *cbs* and *cse* genes are often found co-localized in a single operon (Shatalin et al., 2011). Shatalin et al. showed that in *S. aureus* and *P. aeruginosa*, deletion of the *cse* gene drastically reduced H_2_S biosynthesis and significantly increased antibiotic susceptibility, whereas *cbs* deletion had minimal impact, indicating that CSE is the primary enzyme responsible for H_2_S generation in these two pathogens. In *Vibrio cholerae*, CBS—the enzyme that catalyzes cystathionine biosynthesis from homocysteine and serine—has been most extensively characterized and is established as the central enzyme mediating endogenous H_2_S production (Ma et al., 2021). The highly evolutionarily conserved enzyme 3-MST has been primarily studied in *E. coli*. Research indicates that its H_2_S-synthesizing activity exhibits a pronounced oxygen dependency. Specifically, under aerobic conditions, 3-MST serves as the principal source of endogenous H_2_S, whereas under anaerobic conditions, this role is assumed by cysteine desulfurase (IscS). Consistent with this functional division, genetic deletion of IscS leads to a significant reduction in anaerobic H_2_S production, while deletion of *3-MST* does not produce a comparable effect (Shatalin et al., 2011; Wang et al., 2019a). The relationship between H_2_S and antibiotic susceptibility may therefore be species- and context-dependent, given the considerable variability in the enzymatic sources of H_2_S across different bacteria.

Unlike the mammalian H_2_S biosynthetic machinery, which employ multi-enzyme coordination and elaborate regulation to maintain homeostasis, the bacterial system functions as a highly efficient and inducible survival-defense apparatus (Giedroc, 2017; Pal et al., 2018; Wallace et al., 2018; Nauta and Burton, 2025). This fundamental distinction, especially the pivotal role of H_2_S in antibiotic defense, establishes it as a critical entry point for deciphering bacterial resistance. Thus, elucidating how endogenous H_2_S governs multidrug resistance is of major theoretical and translational importance for developing novel targeted therapies.

This review offers a comprehensive synthesis of bacterial hydrogen sulfide (H_2_S) biosynthesis and its multifaceted roles in modulating antibiotic susceptibility and persistence, encompassing enzymatic pathways, protective mechanisms, and context-dependent effects. It further surveys state-of-the-art detection probes for H_2_S, alongside therapeutic strategies such as scavengers and biosynthesis inhibitors, while delineating the critical challenges and prospective avenues for clinical translation.

## 2 Molecular mechanisms of endogenous H_2_S-mediated multidrug resistance in bacteria

Although not all antibiotics share the same bactericidal mechanism, certain classes—such as fluoroquinolones—disrupt metabolism to trigger a Fenton-mediated superoxide/·OH cascade, causing oxidative damage to biomolecules and DNA double-strand breaks (Gusarov et al., 2009; Kohanski et al., 2010). Endogenous H_2_S serves as a key gaseous signaling molecule in bacteria and plays a central role in their antibiotic stress response. To counteract antibiotic pressure, bacteria employ a multifaceted defense strategy mediated by H_2_S. Specifically, H_2_S can sequester divalent iron to suppress the Fenton reaction and subsequent generation of reactive oxygen species. Furthermore, through formation of persulfide/polysulfide or other reactive sulfane sulfur species, it generates sulfane sulfur species capable of degrading β-lactam antibiotics and activating drug efflux pumps (Kimura, 2011). Additionally, H_2_S modulates cellular energy metabolism to promote a protective dormant state. Collectively, these mechanisms operate synergistically across multiple levels to alleviate antibiotic-induced stress and enhance bacterial survival.

### 2.1 Endogenous H_2_S enhances antibiotic resistance in bacteria by blocking the Fenton reaction

Endogenous H_2_S, a key mediator of bacterial stress adaptation, has been shown to mitigate oxidative damage primarily by chelating intracellular free iron, thereby inhibiting the Fenton reaction. This conclusion is supported by multiple independent studies. (Shatalin et al., 2011). demonstrated that loss of endogenous H_2_S production significantly increases bacterial susceptibility to diverse antibiotics (e.g., quinolones and β-lactams), and that this phenotype is reversible with exogenous H_2_S. Further studies demonstrated that pretreatment of 3-MST-deficient *E. coli* with either the iron chelator 2,2’-dipyridyl or the ROS scavenger thiourea significantly enhanced the strain’s survival rate under gentamicin stress, restoring it to a level comparable to that of the wild-type strain. Notably, subsequent supplementation with NaHS (an exogenous H_2_S donor) exerted no additional protective effect. This further confirms that one of the targets of H_2_S is the iron-dependent Fenton reaction.

**Figure 2:**
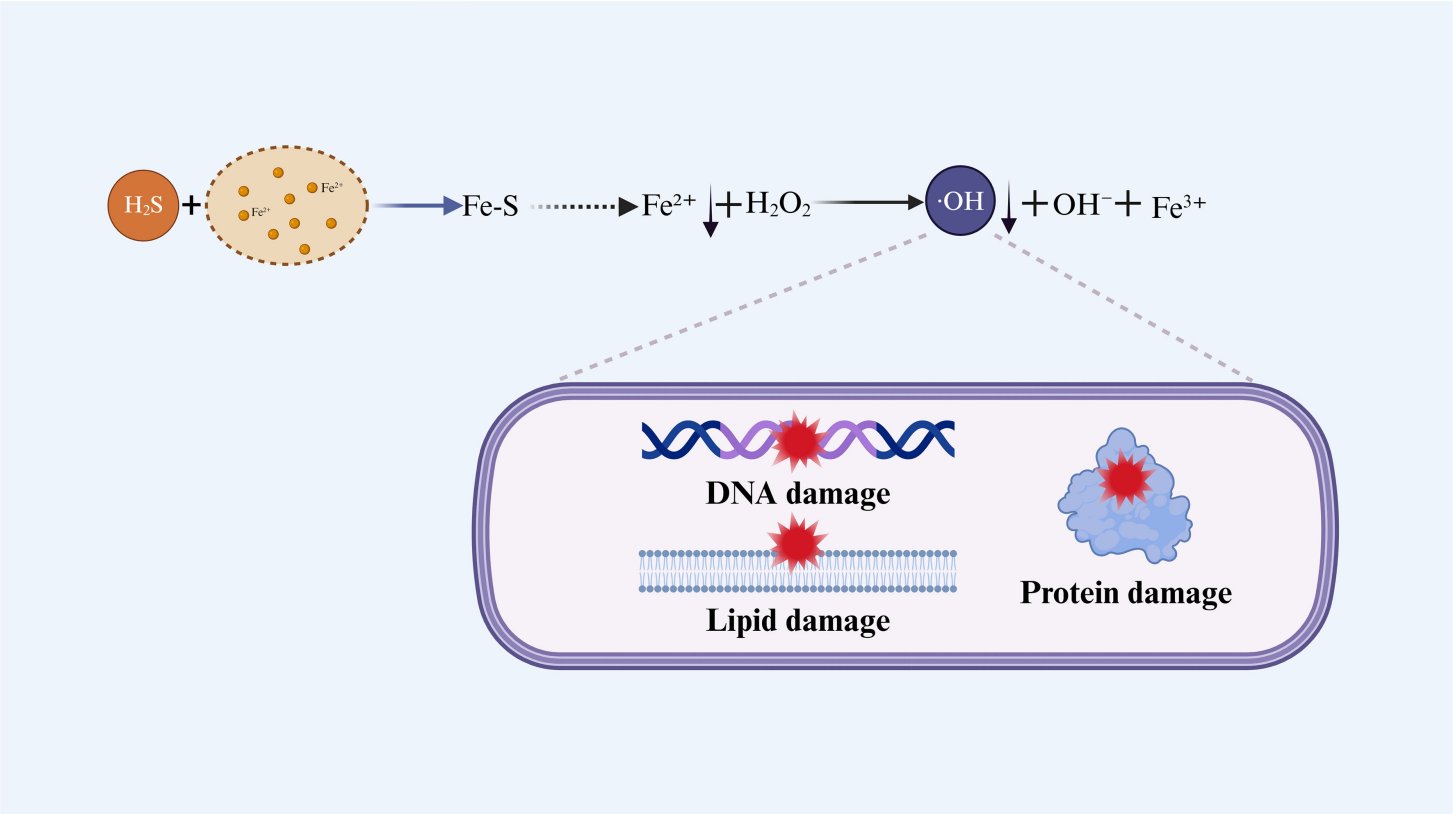
Endogenous H_2_S protects bacterial macromolecules—including DNA, lipids, and proteins—from oxidative damage. This protection is achieved by chelating free Fe²^+^, which blocks the Fenton reaction and thereby suppresses the formation of antibiotic-induced hydroxyl radicals (OH).

Mironov and Nonoyama et al. elucidated the molecular basis by which H_2_S mitigates oxidative DNA damage and preserves genomic integrity in bacteria. Their work demonstrates that H_2_S rapidly chelates intracellular free Fe²^+^, thereby establishing a source-blocking mechanism against the Fenton reaction and the generation of hydroxyl radicals (·OH). In *E. coli* mutants with aberrantly high levels of intracellular free iron, (Mironov et al., 2017). found that H_2_S production was substantially reduced; however, this reduction could be reversed by the addition of an iron chelator. Under conditions of high-iron or H_2_O_2_ stress, H_2_S-overproducing strains exhibited not only a significantly higher induction threshold for the SOS response (a key DNA repair pathway) but also a sharp reduction in DNA double-strand breaks, from >60% to <10%. (Nonoyama et al., 2024). used an iron-sensitive fluorescent probe to dynamically monitor intracellular free Fe²^+^ and directly observed a rapid decrease in the Fe²^+^ signal after exogenous H_2_S treatment. In parallel, H_2_S treatment significantly reduced the proportion of linearized chromosomes—a direct marker of DNA double-strand breaks—from approximately 45% to less than 10%. These studies collectively establish that H_2_S safeguards genomic integrity via a core defense mechanism centered on “iron chelation–Fenton reaction inhibition,” fully elucidating a critical molecular strategy through which bacteria mitigate oxidative stress (**Figure 2**).

### 2.2 The role of sulfane sulfur in H_2_S-induced drug resistance

In addition to alleviating oxidative damage, endogenous H_2_S and its derivatives employ two primary strategies to neutralize drugs, which include direct chemical modification of antibiotics and efflux pump activation.

**Figure 3:**
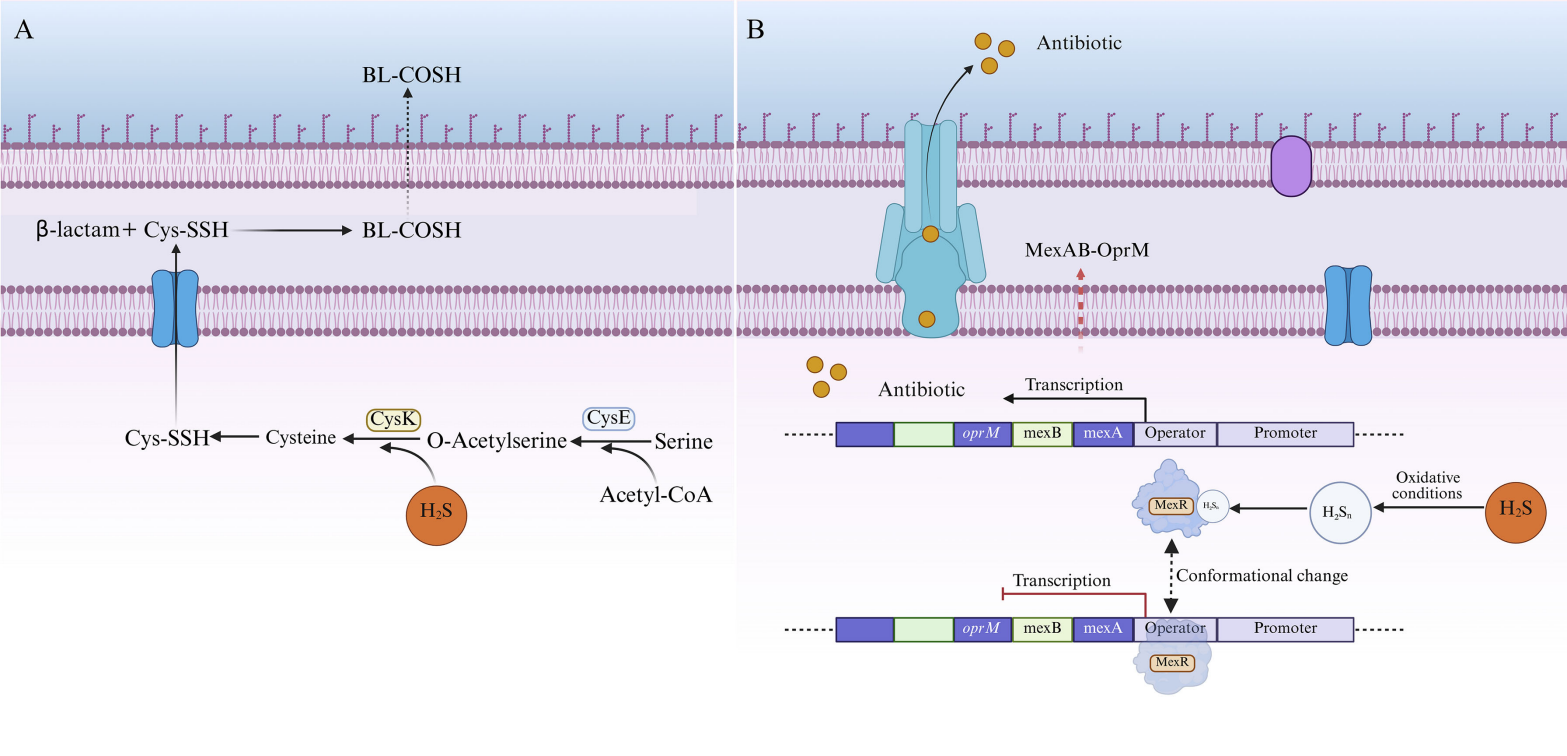
Endogenous H_2_S mediates bacterial antibiotic resistance via two principal mechanisms: direct antibiotic degradation and efflux pump activation. **(A)** Direct antibiotic degradation. In bacteria, H_2_S is converted to cysteine persulfide (Cys-SSH), which acts as a nucleophile to attack the β-lactam ring of antibiotics. This leads to ring opening and formation of the inactivated product BL-COSH, thereby blocking antibiotic binding to penicillin-binding proteins (PBPs). **(B)** Efflux pump activation. H_2_S-derived sulfane sulfur oxidizes key cysteine residues in the MexR repressor. This modification triggers a conformational change in MexR, causing its release from DNA and derepression of the mexAB-oprM operon. Consequently, expression and assembly of the MexAB-OprM efflux pump are enhanced, promoting antibiotic efflux.

The primary mechanism of β-lactam antibiotics involves the specific targeting of penicillin-binding proteins (PBPs) on the bacterial cell wall, thereby disrupting peptidoglycan synthesis (Bush and Bradford, 2016; Lima et al., 2020; Oliveira et al., 2024). (Ono et al., 2021). demonstrated that endogenous H_2_S is converted into cysteine persulfide (CysSSH) in bacterial cells, where it acts as a potent nucleophile to directly cleave the β-lactam ring of β-lactam antibiotics, resulting in their inactivation (**Figure 3A**). Study demonstrated that on agar plates containing CysSSH, the inhibition zones of β-lactam antibiotics (e.g., penicillin G, ampicillin, and meropenem) were markedly reduced or even eliminated compared to CysSSH-free controls, and this effect could not be restored by H_2_S or cystine alone. The CysSSH metabolite thiocarboxylic acid derivative (BL-COSH) was also detected in bacterial cultures by LC-MS/MS analysis.

Drug efflux pumps, which are key membrane protein complexes that mediate bacterial drug resistance, confer antibiotic tolerance by actively expelling antimicrobial agents and thereby reducing their intracellular concentration (Yılmaz and Özcengiz, 2017; Chetri, 2023; Gaurav et al., 2023; Hajiagha and Kafil, 2023). Endogenous H_2_S can be further converted into more reactive sulfane sulfur species, such as glutathione persulfide (GSSH) and inorganic polysulfides (H_2_S_n_, n≥2), under the catalysis of sulfide-quinone oxidoreductase (SQR) (Lü et al., 2017; Li et al., 2019b; Iciek et al., 2023; Koike and Ogasawara, 2024). Xuan et al (Xuan et al., 2020). discovered that sulfane sulfur from H_2_S upregulates the MexAB-OprM efflux pump in *P. aeruginosa*, which exports various antibiotics to lower their intracellular levels and enhance bacterial survival under drug stress (Mbaveng et al., 2015; Tsutsumi et al., 2019) (**Figure 3B**). Mechanistically, they discovered that H_2_S_n_ activates the transcriptional repressor MexR by inducing the formation of a disulfide bond between the conserved Cys^30^ and Cys^62^ residues. This modification reduced the DNA-binding activity of MexR by over 80%, as demonstrated by EMSA, thereby leading to the activation of the MexAB-OprM drug efflux pump system in *P. aeruginosa*.

### 2.3 Endogenous H_2_S-induced bacterial persistence via respiratory chain inhibition

**Figure 4:**
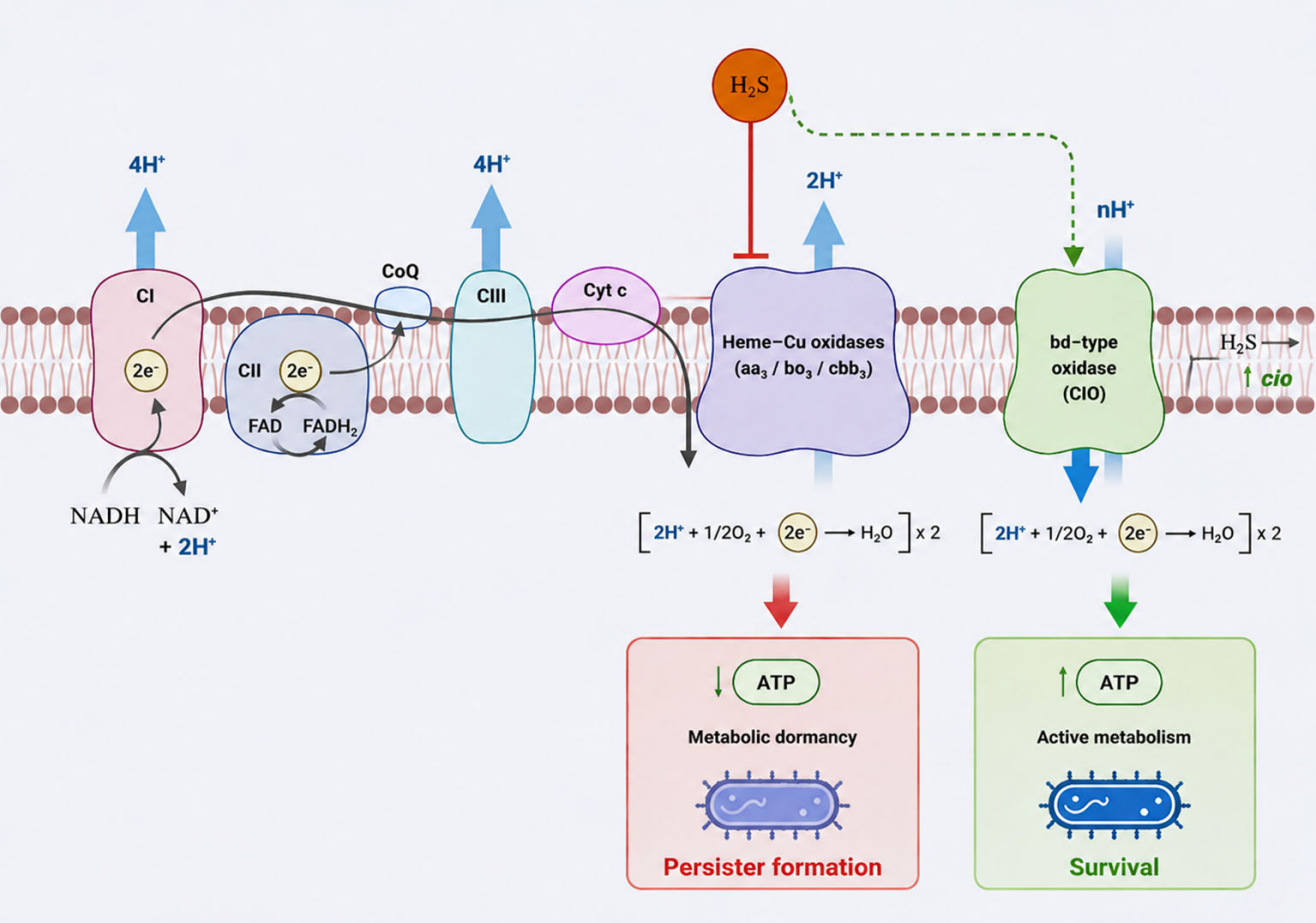
H_2_S triggers the formation of antibiotic-tolerant persister cells by inducing metabolic dormancy via specific inhibition of heme-copper terminal oxidases (e.g.,*aa*3/*bo*3*/cbb*3). This inhibition disrupts electron transfer and proton translocation, leading to impaired ATP synthesis and subsequent intracellular ATP depletion. In contrast, H_2_S does not target *bd*-type oxidase; therefore, it cannot interfere with electron flux or proton pumping through this alternative respiratory pathway, nor does it promote persister cell formation via this route.

Persister cells are a subpopulation of microbial cells that exhibit high tolerance to antibiotics and represent a critical phenotypic state contributing to the persistence and recurrence of chronic infections. Antibiotic tolerance in persister cells is closely associated with entry into a metabolically dormant state. One proposed trigger for this dormancy is endogenous H_2_S, thought to act by inhibiting heme-copper terminal oxidases, an inhibition that has been characterized in detail at both the biochemical and structural levels (**Figure 4**). (Nicholls et al., 2013). determined that H_2_S inhibits cytochrome c oxidase with an exceptionally low inhibition constant (K_i_= 0.45 µM), reflecting potent inhibition at submicromolar concentrations, and a high rate constant (2.2×10^4^ M^-1^·s^-1^), indicating rapid enzyme inactivation, while (Hill et al., 1984) used electron paramagnetic resonance to show that H_2_S directly targets the cytochrome a_3_ and Cu_β_ sites, acting as a reductant that disrupts electron transfer. Consistent with this respiratory blockade, treatment with H_2_S donors causes a rapid collapse of intracellular ATP, with (Shatalin et al., 2011). observing a reduction of over 80% within 30 minutes in *S. aureus*. Extending these observations to persister physiology, the same group later demonstrated that genetic deletion of the H_2_S-producing enzyme CSE, or its pharmacological inhibition, reduced persister counts by 1–2 orders of magnitude in both *S. aureus* and *P. aeruginosa*, whereas exogenous H_2_S supplementation promoted persister formation. Nevertheless, the authors also noted that this regulatory effect is not observed in strains harboring cytochrome *bd* oxidase, underscoring the need to consider the diversity of terminal oxidases in bacterial respiration (Korshunov et al., 2016).

Indeed, as extensively reviewed by (Kaila and Wikström, 2021), bacterial respiratory chains typically harbor multiple terminal oxidases with distinct catalytic properties and differential sensitivities to environmental inhibitors, including various heme-copper oxidases (e.g., aa_3_-, bo_3_-, and cbb_3_-type oxidases) as well as cytochrome bd-type oxidases that lack copper at the active site. This intrinsic redundancy allows bacteria to redistribute electron flux through alternative respiratory branches when one terminal oxidase is compromised. This principle is particularly relevant for cytochrome *bd* oxidases, which exhibit intrinsic resistance to H_2_S. In *E. coli*, (Forte et al., 2016). demonstrated that while the heme-copper bo_3_ oxidase is strongly inhibited by sulfide, cytochrome *bd* oxidases remain active and sustain oxygen consumption and aerobic growth under sulfide stress. Similarly, recent work in *P. aeruginosa* by (Nastasi et al., 2024). showed that the *bd*-type cyanide-insensitive oxidase (CIO) is intrinsically resistant to H_2_S and maintains respiratory activity during sulfide exposure. Moreover, H_2_S induces *cio* expression, whereas *cio*-deficient mutants display increased sulfide sensitivity, indicating that bacteria actively remodel their respiratory machinery to favor sulfide-resistant pathways rather than undergoing obligatory respiratory shutdown.

In light of this respiratory flexibility, the connection between H_2_S exposure, metabolic dormancy, and persister formation requires further experimental validation. Therefore, the proposed model should be interpreted as a possible hypothesis—or a consequence of metabolic adaptation under specific physiological contexts—rather than as an established universal mechanism linking H_2_S-mediated respiratory inhibition to persistence.

## 3 Fluorescent probes for H_2_S with diverse mechanistic strategies

H_2_S functions as a critical gaseous signaling molecule in numerous physiological and pathological processes. Consequently, the development of fluorescent probes with high selectivity and sensitivity for specific detection and real-time tracking of H_2_S has emerged as a significant research focus in the fields of chemical biology and biomedical analysis (**Table 1**). Importantly, although many H_2_S fluorescent probes were originally developed in mammalian systems, only a subset has been validated in bacterial systems, particularly under antibiotic stress conditions. This is due to differences in H_2_S concentration ranges, cellular permeability barriers, and intracellular thiol environments between mammalian and bacterial systems, which critically influence probe performance. This section addresses hydrogen sulfide probes with documented applications in bacterial infection; other probes lacking such applications are summarized only in **Table 1**.

**Table 1:** Fluorescent probes for hydrogen sulfide with diverse mechanistic strategies.

Probe name	Fluorophore scaffold	H_2_S-responsive group	Mechanism of action	Application in bacteria	Ref.
SF series	Rhodamine	Azide	Reduction of azide to amine	+	(Lippert et al., 2011)
MeRho-Az	Methyl rhodol	Azide	Reduction of azide to amine	–	(Hammers et al., 2015)
AzMC	Coumarin	Azide	Reduction of azide to amine	+	(Olson et al., 2019)
NATRP	Naphthalimide	Azide	Reduction of azide to amine	+	(Gupta et al., 2025)
WSP-5	Fluorescein	Azide	Reduction of azide to amine	+	(Zhou et al., 2022)
Pyridine N-oxide probes	near-IR dyes	Pyridine N-oxide	Reduction of N-oxide to amine	–	(Wu et al., 2014)
Nitroso/oxime probes	Coumarin	Nitroso/Oxime	structural rearrangement	–	(Dong et al., 2020)
WSP1	Fluorescein	S–S	hydropersulfide intermediate → cyclization	+	(Liu et al., 2011)
BP-PS	BODIPY	Aryl boronate	H_2_S-induced nucleophilic substitution and fluorophore release	+	(Li et al., 2019a)
SeSP series	BODIPY, ICT-, or PET-based	Se–S	Se elective reaction with H_2_S	–	(Wang et al., 2019b)
SeP1/2	Rhodamine,	Se–Se	H_2_S-mediated reductive cyclization of the diselenide switch	–	(Peng et al., 2015)
DNP/DNBS	Fluorescein, Rhodamine, Coumarin	DNP/DNBS	H_2_S nucleophilic substitution	+	(Cao et al., 2012) (Fu et al., 2014)
HD-WY	Fluorescein, Rhodamine, Coumarin	DNP/DNBS	H_2_S-triggered DNP cleavage and fluorescence recovery	+	(Zhou et al., 2026)
HS-CL	Fluorescein, Rhodamine, Coumarin	DNP/DNBS	H_2_S-induced nucleophilic substitution and cleavage	+	(Yang et al., 2025)
NBD-piperazine	Coumarin, Fluorescein derivatives	NBD-amine linkage	H_2_S-induced cleavage	+	(Montoya et al., 2013)
Sulfane sulfur probes	Coumarin, cyanine-based	Dithiocarbamates, thioesters, thiophosphates, etc.	Electrophilic trapping or thiol-exchange/cyclization	+	(Liu et al., 2014)
HSip-1	Fluorescein	Cu²^+^-chelating structure	metal displacement	–	(Sasakura et al., 2011)
Zn²^+^-displacement probes	Fluorescein	Zn²^+^-chelating ligand	metal displacement	–	(Tang et al., 2013)
Cd²^+^-displacement probes	Rhodamine	Cd²^+^-chelating ligand	metal displacement	–	(Takashima et al., 2014)

### 3.1 Probes based on the reduction of oxidized nitrogen functionalities

**Figure 5:**
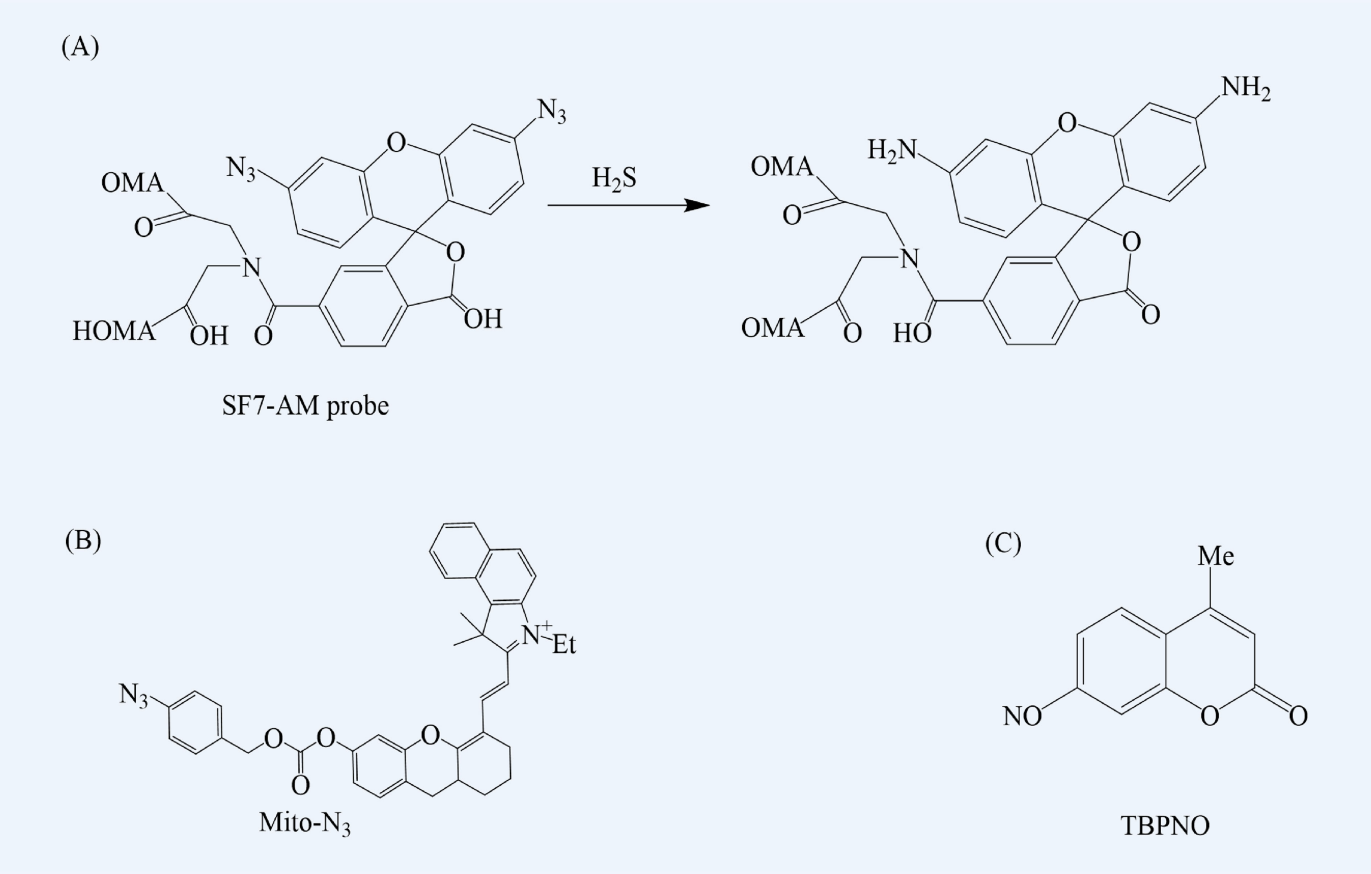
**(A)** Chemical structure of the SF7-AM probe and its reaction mechanism toward H_2_S. **(B)** Chemical structure of the mitochondria-targeted azide probe Mito-N_3_. **(C)** Chemical structure of the small-molecule H_2_S fluorescent probe TBNNO.

H_2_S fluorescent probes based on the reduction of oxidized nitrogen functionalities represent the earliest systematically developed and still the most widely used class of reaction-based probes (**Figure 5**). Their core design principle exploits the reducing property of H_2_S to selectively reduce nitrogen-containing groups such as azide (–N_3_) and nitro (–NO_2_), thereby relieving fluorescence quenching and achieving a turn-on signal response (Henthorn and Pluth, 2015). Exemplified by the SF series of rhodamine-azide probes from the Chang group (Lippert et al., 2011) and the methyl rhodol-based MeRho-Az probe reported by the Pluth group (Hammers et al., 2015), this system has evolved into a versatile platform for constructing advanced H_2_S probes, including targeted, ratiometric, two-photon, and even chemiluminescent variants, owing to its excellent selectivity, significant fluorescence enhancement, and good biocompatibility (Bae et al., 2013; He et al., 2015; Liu et al., 2018; Levinn and Pluth, 2021). Among these, the WSP series (particularly WSP-5) have been extensively employed in bacterial systems for visual detection of endogenous H_2_S in Gram-negative bacteria, notably for monitoring dynamic changes under oxidative stress and antibiotic pressure, thereby bearing direct relevance to studies on antibiotic tolerance mechanisms (Zhou et al., 2022). Concurrently, 7-azido-4-methylcoumarin (AzMC), as a well-established small-molecule azide probe, has been widely utilized for bacterial H_2_S sensing, attributable to its simple structure, fast response, and suitability for microbial imaging. More recently, naphthalimide-based azide probes, such as NATRP, have further expanded the application of this strategy toward bacterial imaging (Gupta et al., 2025). NATRP has been validated for real-time fluorescence imaging of endogenous H_2_S in living bacterial cells, demonstrating its potential for monitoring bacterial H_2_S-related physiological processes at the cellular level.

### 3.2 Electrophilic H_2_S fluorescent probes

**Figure 6:**
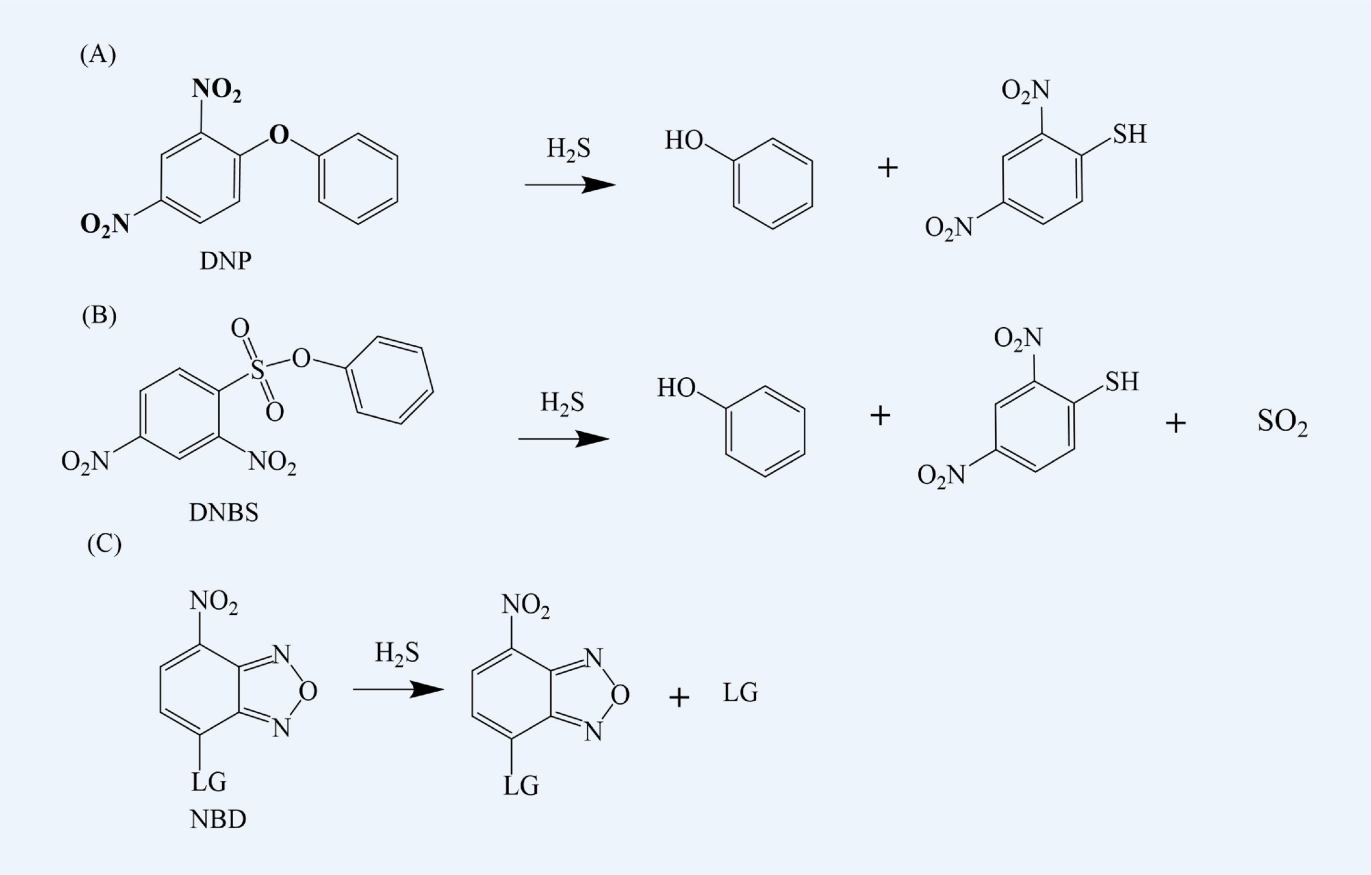
**(A–C)** Representative nucleophilic substitution–based mechanisms for H_2_S fluorescent probes.

Electrophilic H_2_S fluorescent probes function by exploiting the strong nucleophilicity of H_2_S/HS^-^ (Fosnacht and Pluth, 2024). These probes incorporate designed electrophilic centers that undergo addition, substitution, or bond-cleavage reactions upon H_2_S attack, leading to measurable changes in fluorescence (**Figure 6**). Common electrophilic triggers include activated alkenes (Michael acceptors), aryl halides, the 2,4-dinitrophenyl (DNP/DNBS) group, NBD derivatives, as well as thioesters, dithiocarbamates, and thiophosphates (Maeda et al., 2005; Qian et al., 2011; Cao et al., 2012; Liu et al., 2012; Zhang et al., 2013; Bae et al., 2014; Karakuş et al., 2016). The reaction of H_2_S with these sites typically disrupts photoinduced electron transfer (PET) or intramolecular charge transfer (ICT) within the fluorophore, resulting in a turn-on or ratiometric fluorescence response.

Recently developed probes HD-WY and HS-CL have further broadened the application of fluorescent probes to bacterial environments. Specifically, HD-WY employs 2, 4-dinitrophenyl (DNP) as a recognition moiety conjugated to a hemicyanine fluorophore; its mechanism operates via H_2_S-triggered nucleophilic aromatic substitution, liberating a fluorescent species. This probe has been explicitly applied for *in situ* imaging of H_2_S in *Helicobacter pylor*i-induced gastritis models (Zhou et al., 2026). In contrast, HS-CL integrates an electrophilic recognition motif into a fluorescent scaffold, enabling selective fluorescence activation upon H_2_S exposure and rendering it suitable for monitoring endogenous H_2_S dynamics within bacterial cells. The primary utility of this probe lies in the rapid detection of antibiotic-resistant bacteria, and its efficacy has been systematically validated against a panel of standard strains, including *Acinetobacter baumannii*, methicillin-resistant *S. aureus*, *E. coli*, and *Klebsiella pneumoniae*, as well as clinical isolates (e.g., *E. coli*, *K. pneumoniae*, and *P. aeruginosa*) derived from patients (Yang et al., 2025).

### 3.3 Strategies for fluorescent probes targeting sulfane sulfur

**Figure 7:**
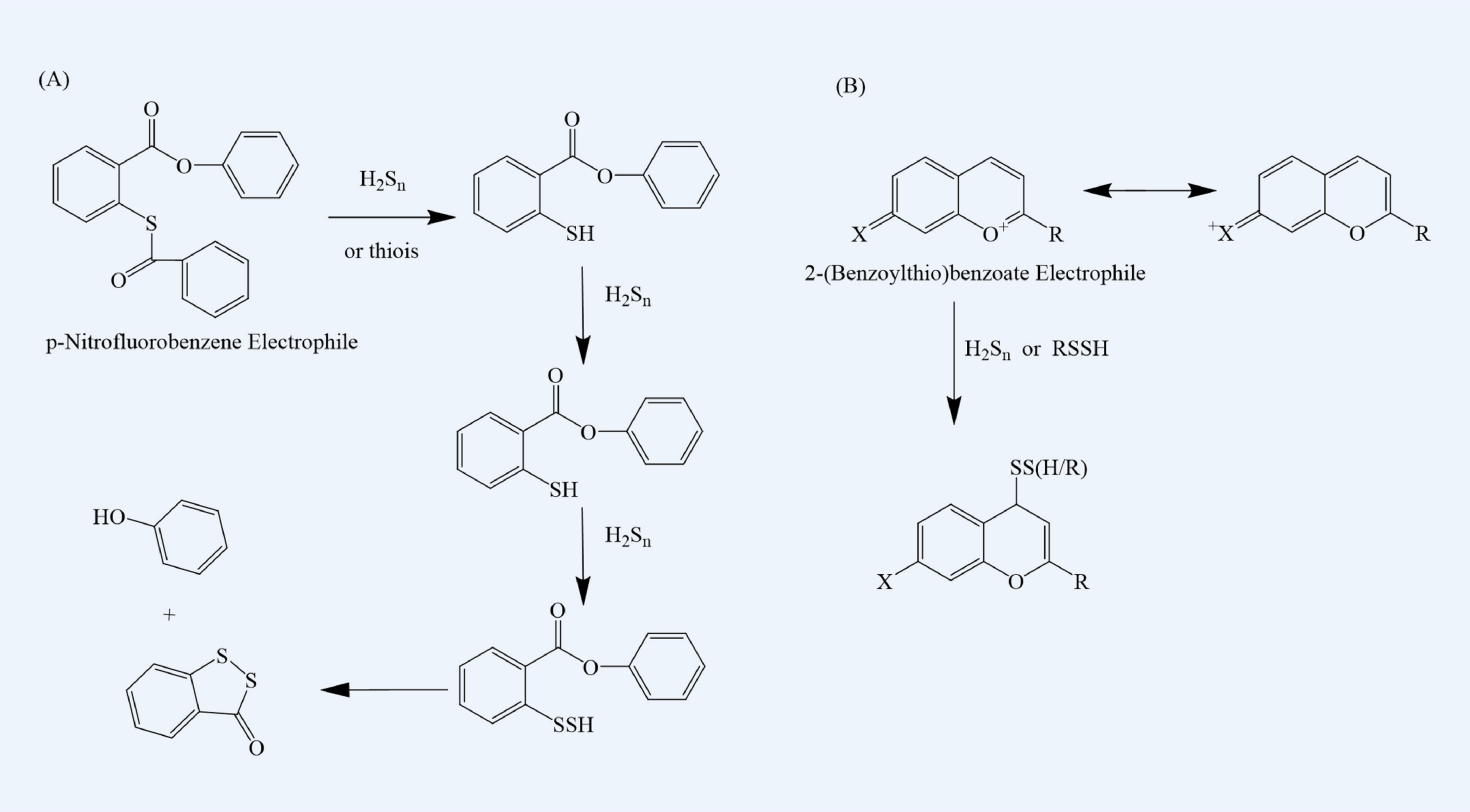
**(A, B)** Representative nucleophilic reaction mechanisms of sulfane sulfur species–responsive fluorescent probes.

Sulfane sulfur species—characterized by sulfur in the zero or +1 oxidation state (S^0^/S^+^)—include polysulfides (H_2_S_n_), persulfidated proteins or small molecules (RSSH), polysulfide chains (RSS_n_R), and elemental sulfur (S_8_). These high-valent sulfur compounds play key roles in cellular redox regulation and protein sulfuration (Filipovic et al., 2018; Kolluru et al., 2020; Pedre et al., 2021). Fluorescent probes designed to detect them typically exploit the strong nucleophilicity of their terminal sulfur atoms and their distinct sulfur-chain chemistry, enabling selective sensing via electrophilic trapping or thiol-exchange/cyclization mechanisms (Benchoam et al., 2020) (**Figure 7**).

Electrophilic trapping probes incorporate sites such as dithiocarbamates, activated thioesters, thiophosphates, or Michael acceptors (Liu et al., 2014; Chen et al., 2015; Hou et al., 2016; Kawagoe et al., 2017). Sulfane sulfur attacks these electrophilic centers to form polysulfide adduct or undergo thiol exchange, resulting in fluorescence dequenching or fluorophore release. In contrast, thiol-exchange/cyclization probes use triggers like benzodithiolone, thioamide, or designed disulfide linkers (Li et al., 2023). Reaction with sulfane sulfur induces cyclization to stable sulfur-containing heterocycles, producing turn-on or ratiometric fluorescence signals (Benchoam et al., 2020). Among these diverse probes, BP-PS is a bioluminescence imaging probe characterized by both high specificity and high sensitivity, which permits the direct detection of endogenous hydrogen polysulfides (H_2_S_n_) in the absence of an external excitation light source (Li et al., 2019a). By eliminating autofluorescence interference from tissues, this approach ensures a superior signal-to-noise ratio. The capability of the BP-PS probe for real-time visualization of endogenous H_2_S_n_ has been validated in living cells and in murine models of bacterial infection.

## 4 Targeting bacterial H_2_S biosynthesis with inhibitors

In bacteria, endogenous H_2_S functions as a gaseous signaling molecule that critically promotes antibiotic tolerance by modulating redox homeostasis, inducing metabolic dormancy, and facilitating persister cell formation. Therefore, targeting the endogenous H_2_S biosynthesis pathway emerges as a promising strategy to combat antibiotic resistance and eliminate persister cells. A key strategy in this progress involves designing inhibitors that target the pyridoxal phosphate (PLP)-binding site of critical H_2_S-producing enzymes, including CSE and CBS, thereby suppressing their activity and inhibiting H_2_S production (Singh and Banerjee, 2011; Wang, 2012; Asimakopoulou et al., 2013; Gregory et al., 2016; Yadav et al., 2019). Given that PLP is an essential cofactor in critical human physiological processes such as amino acid metabolism and neurotransmitter synthesis (Percudani and Peracchi, 2003; Amadasi et al., 2007; Fitzpatrick et al., 2007; Cellini et al., 2014), strategies targeting it carry a high risk of off-target effects and consequent toxicity. To overcome these limitations, a promising alternative is the development of novel, highly selective inhibitors that act on H_2_S synthases through PLP-independent mechanisms, an area of increasingly prominent research interest (**Table 2**).

**Table 2:** Representative inhibitors targeting bacterial H_2_S biosynthesis and their characteristics.

Inhibitor	Target	Mechanism of action	Effect	Ref.
EGCG	CBS	Non-PLP-dependent inhibition via substrate channel blockade	Exhibiting high binding affinity (IC_50_ 0.12 μM, Kd 0.14–0.6 μM)	(Zuhra et al., 2022)
NL series	CSE	Highly selective bacterial CSE inhibitor from structure-based design	Inhibiting CSE at Sub-µM with Broad Synergy	(Shatalin et al., 2021)
Pioglitazone	3-MST	Competitive inhibition through binding key active site residues	Selective 3-MST inhibition with 3-fold ROS increase at 400 µM	(Croppi et al., 2020)
H_2_S scavenger	H_2_S molecule	Chemically quenches H_2_S to lower intracellular levels	Attenuating H_2_S-mediated antioxidant protection.	(Sun et al., 2024)
Metabolic intervention	CysE/CysM	Limiting the common precursor pool for H_2_S and glutathione synthesis	H_2_S downregulation restores macrolide bactericidal efficacy	(Wu et al., 2022)

### 4.1 EGCG as a highly selective and PLP-independent CBS inhibitor

Zuhra et al (Zuhra et al., 2022). performed high-throughput screening to identify the green tea polyphenol epigallocatechin gallate (EGCG) as a potent and selective inhibitor of CBS, exhibiting an IC_50_ of 0.12 μM. Microscale thermophoresis confirmed that EGCG binds directly to CBS with sub-micromolar affinity, demonstrating a dissociation constant (Kd) in the range of approximately 0.14 to 0.6 μM. Spectroscopic studies revealed that EGCG inhibits CBS not by interacting with the catalytic cofactor PLP, but by occluding the substrate channel to the PLP-active site. This cofactor-independent mechanism thus preferentially targets H_2_S signaling and consequently positions EGCG as a promising therapeutic candidate.

Although EGCG exhibits strong biochemical potency and selectivity, its pharmacokinetic limitations, including low oral bioavailability, rapid metabolic degradation, and poor stability, may restrict its translational potential as a CBS inhibitor. Therefore, structural optimization, formulation strategies, or the development of more stable EGCG-derived analogues may help improve its drug-like properties while preserving CBS inhibitory activity (Zhang et al., 2024).

### 4.2 NL-series inhibitors for selective bacterial CSE inhibition

Using structure-based design and virtual screening, Shatalin et al. developed a series of novel small-molecule inhibitors (termed NL1, NL2, and NL3) against the H_2_S-producing enzyme CSE. The NL-series inhibitors exhibited significantly greater potency against CSE from *S. aureus* and *P. aeruginosa* (IC_50_ in the sub-micromolar range) than against the human ortholog. *In vitro*, the lead compound NL1 markedly enhanced the efficacy of aminoglycoside, fluoroquinolone, and β-lactam antibiotics, reducing both MIC and MBC by 2- to 15-fold. Furthermore, *in vivo* studies demonstrated that a sub-therapeutic dose of NL1 synergized with gentamicin, increasing survival from 10% to 50% in a murine *S. aureus* sepsis model and reducing bacterial load by ~100-fold in a *P. aeruginosa* pneumonia model. These findings thus highlight the potential of inhibiting the bacterial H_2_S pathway to counteract antibiotic resistance.

Nevertheless, these investigations remain at the preclinical stage, and the pharmacokinetic properties, stability, biodistribution, and long-term safety profile of NL inhibitors have yet to be comprehensively characterized (Shatalin et al., 2021). Their antimicrobial efficacy has been predominantly validated in *S. aureus* and *P. aeruginosa*, whereas their broad-spectrum activity against other clinically relevant pathogens remains uncertain. Accordingly, further studies are warranted to delineate their therapeutic window and assess their clinical potential.

However, these studies are still preclinical. The pharmacokinetics, stability, distribution, and long-term safety of NL inhibitors are not fully known. Their activity has mainly been shown in *S. aureus* and *P. aeruginosa*, and broader activity against other pathogens is unclear. More work is needed to define their therapeutic window and evaluate their clinical potential.

### 4.3 Pioglitazone repurposed as a selective bacterial 3-MST inhibitor

In specific bacterial species, 3-MST represents an additional key source of endogenous H_2_S, alongside the canonical enzymes CSE and CBS. Giorgia et al (Croppi et al., 2020). identified the thiazolidinedione antidiabetic drug Pioglitazone as a potent competitive inhibitor of 3-MST through high-throughput screening. This compound specifically binds to the Arg178 and Ser239 residues in the active site of *E. coli* 3-MST, inhibiting the conversion of the 3-mercaptopyruvate substrate, while exhibiting almost no effect on human and mammalian 3-MST. Further investigation demonstrated that pioglitazone (400 µM) inhibits eMST activity, thereby disrupting the bacterial redox balance and increasing intracellular reactive oxygen species (ROS) levels in *E. coli* by approximately threefold, an elevation that was reversible upon supplementation with an exogenous H_2_S donor (Hanefeld, 2001). Collectively, these results position pioglitazone as a valuable chemical probe for elucidating bacterial H_2_S physiology, highlighting the broader promise of drug repurposing for novel anti-infective development.

Although pioglitazone can inhibit the activity of bacterial 3-MST, its IC_50_ is far above clinically achievable plasma drug concentrations (Croppi et al., 2020). Therefore, it is necessary to further enhance its inhibitory activity through structural optimization or the development of more potent derivatives, and to systematically evaluate the clinical feasibility of targeting bacterial 3-MST as an anti-infective therapeutic strategy.

### 4.4 Reduction of intracellular H_2_S via scavenging and biosynthesis inhibition

In addition to direct H_2_S synthase targeting, researchers have explored other innovative ways to intervene in the H_2_S pathway. For example, (Sun et al., 2024). developed an H_2_S-specific scavenger (compound 7b) that chemically quenches H_2_S, directly reducing its intracellular levels and thus impairing the associated antioxidant protection. (Wu et al., 2022). proposed a “source-restricting” metabolic intervention strategy, demonstrating that exogenous supplementation of L-serine significantly downregulates the activity of key enzymes in the cysteine *de novo* synthesis pathway of *Streptococcus suis*—serine acetyltransferase (CysE) and cysteine synthase (CysM)—via a feedback inhibition mechanism. This metabolic reprogramming reduces the biosynthetic precursors of H_2_S and its downstream antioxidant GSH at the source, effectively lowering bacterial H_2_S levels and successfully restoring the bactericidal efficacy of macrolide antibiotics under the tested conditions.

## 5 Discussion

Although the mechanistic basis of H_2_S as a gaseous signaling molecule has been extensively elucidated in mammals, its functional repertoire in bacteria remains comparatively underexplored. Present insights into CSE function are predominantly informed by research on model strains such as *S. aureus* and *P. aeruginosa*. Nevertheless, the question of whether a conserved functional hierarchy exists among CBS, CSE, and 3-MST across diverse bacterial taxa remains unresolved. Notably, in clinically significant pathogens like *S. pneumoniae*, *M. tuberculosis*, and *A. baumannii*, the principal enzyme(s) governing H_2_S biosynthesis still await definitive identification via systematic genetic screening and subsequent functional validation. Moreover, the upstream signaling network through which bacteria sense antibiotic stress to regulate H_2_S production remains poorly defined, leaving the broader regulatory framework controlling H_2_S in bacteria largely elusive. Mapping the complete regulatory pathway is of significant importance for predicting the evolution of drug resistance and developing targeted intervention strategies. In addition, while H_2_S has been suggested to promote persister cell formation through respiratory chain inhibition, it should be recognized that bacterial respiratory systems are highly branched and contain multiple terminal oxidases with distinct sensitivities to sulfide. Hence, inhibition of cytochrome c oxidase alone cannot be considered a universal mechanism for H_2_S-induced dormancy and persistence; rather, respiratory flexibility, alternative terminal oxidases, and adaptive remodeling of electron transport pathways during sulfide exposure must be taken into account. Finally, while host cells produce H_2_S for immune modulation during bacterial infection, the crosstalk between host and pathogen H_2_S systems remains poorly understood. Elucidating this interaction is crucial for a comprehensive understanding of infection pathology.

Although substantial evidence suggests that H_2_S can attenuate the bactericidal effects of antibiotics through various mechanisms, emerging evidence indicates that the impact of H_2_S on antibiotic susceptibility is highly context-dependent. For example (Caruso et al., 2024), found that in *P. aeruginosa*, neither significant reduction nor excessive accumulation of H_2_S production altered the MICs of Carbapenems, Aminoglycosides, and Fluoroquinolones. Moreover, analysis of 100 clinical isolates revealed that multidrug-resistant strains produced significantly lower H_2_S levels than susceptible strains, with a further decline during chronic infection. These observations do not contradict earlier findings but rather highlight that the relationship between H_2_S biology and antibiotic susceptibility depends on bacterial species, physiological context, and experimental conditions. In contrast to the function of endogenous H_2_S, research indicates that the administration of exogenous H_2_S can resensitize bacteria to antibiotics. This is demonstrated by the effective reversal of resistance in non-H_2_S-producing pathogens like *Aeromonas caviae* and *P. guariconensis* when treated with a combination of antibiotics and H_2_S donors such as NaHS. This resensitization process primarily involves inducing a burst of reactive oxygen species (ROS), inhibiting the antioxidant enzyme system (including SOD, CAT, and GSH), interfering with energy metabolism (such as reducing LDH activity), and disrupting cellular structure, ultimately restoring bacterial sensitivity to antibiotics (Selvakumar et al., 2024; 2025). These context-dependent effects elucidate the potential dual role of H_2_S, suggesting its action in bacterial stress responses is contingent upon both concentration and the specific infection milieu. Thus, they provide a novel conceptual framework for designing antibiotic adjuvants that target gas signaling pathways.
